# Highly efficient and thermally stable cyan-emitting ZnS/ZnO phosphors for full-visible-spectrum LED lighting†

**DOI:** 10.1039/d4ra08038f

**Published:** 2025-01-07

**Authors:** Do Quang Trung, Manh Trung Tran, Nguyen Van Du, Nguyen Duy Hung, Tong Thi Hao Tam, Nguyen Tu, Pham Minh Tri, Hoang Gia Chuc, Nguyen Duc Trung Kien, Nguyen Van Quang, Pham Thanh Huy

**Affiliations:** a Faculty of Fundamental Sciences, Phenikaa University Yen Nghia, Ha-Dong District Hanoi 10000 Vietnam trung.doquang@phenikaa-uni.edu.vn; b Faculty of Materials Science and Engineering, Phenikaa University Yen Nghia, Ha-Dong District Hanoi 10000 Vietnam trung.tranmanh@phenikaa-uni.edu.vn; c Faculty of Electronic Materials and Components, School of Materials Science and Engineering, Hanoi University of Science and Technology (HUST) No. 01 Dai Co Viet Hanoi 10000 Vietnam; d Faculty of Information Technology, College of Technology, National Economics University (NEU) 207 Giai Phong Street Hanoi 10000 Vietnam; e Faculty of Electrical and Electronic Engineering, Phenikaa University Yen Nghia, Ha-Dong District Hanoi 10000 Vietnam; f Department of Chemistry, Hanoi Pedagogical University 2 Phuc Yen Vinh Phuc Vietnam

## Abstract

Near-ultraviolet (NUV)-pumped white light-emitting-diodes (WLEDs) often suffer from poor color rendering in the 480–520 nm range, highlighting the need for an efficient cyan phosphor with strong absorption at 370–420 nm. This study presents the successful synthesis of cyan-emitting ZnS/ZnO phosphors using a high-energy planetary ball milling method followed by post-annealing. The fabricated phosphors, with particle sizes ranging from 1 to 3 μm, exhibit strong cyan emission with CIE chromaticity coordinates of (0.2302, 0.3759) and excellent thermal stability with an activation energy of 0.26 eV. A prototype near-ultraviolet (NUV)-pumped cyan LED was developed, achieving chromatic coordinates of (0.2769, 0.4380) and a quantum efficiency of 77% by coating an NUV chip at 370 nm with the synthesized phosphor. These results demonstrate the potential of ZnS/ZnO-based materials as efficient, non-toxic alternatives to rare-earth phosphors, paving the way for advancements in full-spectrum white LEDs for solid-state lighting.

## Abstract 2

Optical properties of ZnS/ZnO phosphor and demonstration of NUV-driven phosphor-coated WLEDs.

## Graphical abstract

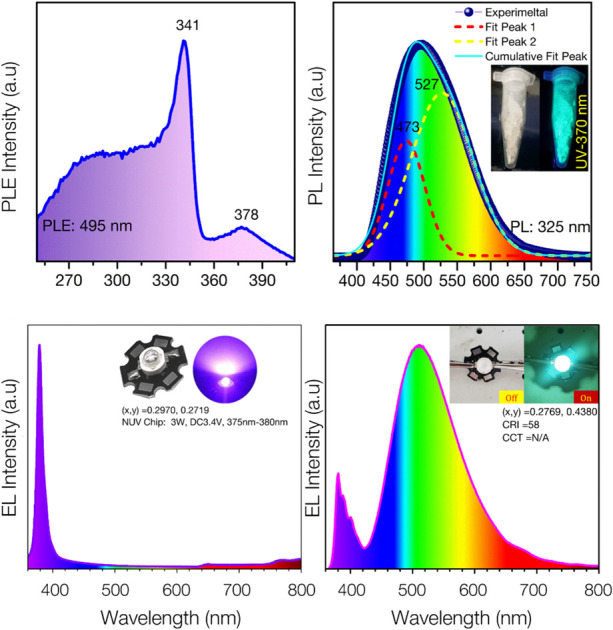

## 1. Introduction

Due to their exceptional optical properties, rare-earth-doped inorganic phosphors have been extensively researched and developed for a wide range of applications, including white light-emitting diodes (WLEDs), display screens, indoor plant cultivation, and biomedical uses.^1^ Commercial WLEDs are generally fabricated by using a blue LED chip (InGaN) coated by a yellow YAG:Ce^3+^ phosphor;^1,2^ however, they only exhibit a low quality of white light (a poor color rendering index, CRI, <75 and high correlated color temperature, CCT, >4500 K) due to lack of red emission.^2^ In addition, compared to incandescence and sunlight, the emission spectra of WLEDs lack a cyan emission (470–500 nm) between the primary blue LED chip and the YAG:Ce^3+^ phosphor emission.^3,4^ Recently, many efforts have been made to enhance the CRI values and adjust the CCT of YAG:Ce^3+^-based WLEDs by adding red phosphors, and a high CRI of around 90 has been achieved.^5^ On the contrary, little attention has been paid to fulfilling the cyan gap by discovering cyan-emitting phosphors well excited by blue light for ultrahigh-CRI (>95) pc-WLEDs.^6^ Another way to make full-spectrum WLEDs with high-CRI could be based on a combination of a near-ultraviolet (NUV) LED chip with red, green, and blue (RGB) or cyan and red phosphor.^7,8^ Many studies have recently been made to find efficient phosphors for NUV-pumped WLEDs. Among them, the research on new phosphors such as high-efficiency cyan emitting phosphors excitable by NUV light is rapidly increasing and has become one of the hot research topics.^2,9^ High-efficiency cyan emitting phosphors generally were synthesized by using rare-earth ions activated by several host lattices, such as Ba_3_Y_2_B_6_O_15_:Ce^3+^, Tb^3+^,^1^ RbBa_2_(PO_3_)_5_: Eu^2+^,^7^ Ba_9_Y_2_Si_6_O_24_:Eu^2+^,^10^ RbNa(Li_3_SiO_4_)_2_:Eu^2+^,^11^ Ba_9_(Lu_2−*x*−*y*_Al_*x*_)Si_6_O_24_:*y*Ce^3+^,^4^ Ca_3_SiO_4_(Cl,Br)_2_:Eu^2+^,^12^ BaZrSi_3_O_9_:Eu^2+^,^8^*etc.* However, these phosphors contain rare-earth and complex components, making them expensive and difficult to synthesize.

As widely known, wide bandgap semiconductors such as zinc oxide (ZnO) and zinc sulfide (ZnS), featuring substantial bandgaps of approximately 3.72–3.77 eV for ZnS and around 3.37 eV for ZnO, hold significant promise as efficient and non-toxic alternatives to rare-earth-based phosphors in applications such as fluorescent and solid-state lighting.^13–16^ Both ZnO and ZnS, when incorporating intrinsic defects, exhibit strong emission across the entire visible spectrum, contingent upon the specific defect type formed. Undoped ZnO usually emits green light associated with oxygen vacancies (V_O_) or blue light related to the zinc interstitials (Zn_i_) defect.^17,18^ In parallel, ZnS exhibits robust emission in the blue spectrum, primarily through the sulfur vacancies defect (V_S_).^19–21^ Recent advancements in ZnS and ZnO-based materials include the successful development of warm-white light-emitting diodes (WLEDs),^22,23^ highlighting the potential of cyan-emitting ZnS/ZnO phosphors. Although, ZnS/ZnO nanocomposite phosphor was fabricated,^24^ microsized ZnS/ZnO phosphors for WLED applications have not been studied yet.

High-energy planetary ball milling has recently emerged as an effective method for synthesizing phosphors at lower calcination temperatures than conventional solid-state reactions, while also introducing defect-related states that create electron/hole traps within the band gap.^25^ These new defect states can result in additional emission peaks in the photoluminescence spectra. Consequently, high-energy planetary ball milling shows great promise for synthesizing cyan-emitting ZnS/ZnO-based phosphors by creating these new defect states.

Herein, cyan-emitting ZnS/ZnO phosphors were synthesized using the high-energy planetary ball milling method coupled with post-annealing. The crystal structure and optical performances of the obtained phosphors were characterized in detail. Furthermore, a cyan LED coated with ZnS/ZnO phosphor prototype was successfully fabricated to demonstrate the potential for full-spectrum white LED applications.

## 2. Experimental

Zinc sulfide (ZnS, 99.99%) and zinc oxide (ZnO, 99.99%) powders from Sigma-Aldrich were used as received. ZnS, ZnO, and their 1 : 1 weight mixture were ball-milled at 500 rpm for 2 hours using a Fritsch Pulverisette7 (Germany), followed by annealing in an argon atmosphere at 1000 °C for 2 hours to evaluate their characteristics.

The crystalline structure was characterized by X-ray diffraction (XRD, Rigaku Smartlab, Japan D2 Phaser A26-X1, Bruker, Germany) with a Cu Kα (*λ* = 0.154 nm) performing at 40 mA tube current. The surface morphologies and compositions were observed by a field emission scanning electron microscopy system (FESEM-JEOL/JSM-7600F) equipped with energy-dispersive X-ray spectroscopy (EDX). The Raman spectra were obtained by a Raman microscopy – XploRA Plus instrument (Horiba, France) using the wavelength of 532 nm of the laser source. The XPS spectra were investigated by an X-ray photoelectron Spectroscope (XPS, VG Scientific, ESCALAB250) radiated by a monochromatic Al Kα source. The UV-vis absorption spectra were obtained by a single-monochromator Jasco V-750 spectroscopy. Photoluminescence (PL) and photoluminescence excitation (PLE) spectra were studied by a spectrofluorometer excited by a 450 W xenon discharge lamp (Nanolog, HORIBA Jobin Yvon). The optical characteristics of LED devices were measured by a spectroradiometer (RadOMA, Gamma Scientific GS-1290).

Finally, a 4 : 1 mixture of polydimethylsiloxane (PDMS, Dow Corning OE-6370) and ZnS/ZnO phosphor was prepared to fabricate WLED prototypes. The mixture was coated onto a 370 nm UV chip, and then cured in an oven (ASONE AVO-310SB-D) at 150 °C for 4 hours.

## 3. Results and discussion

**Fig. 1:**
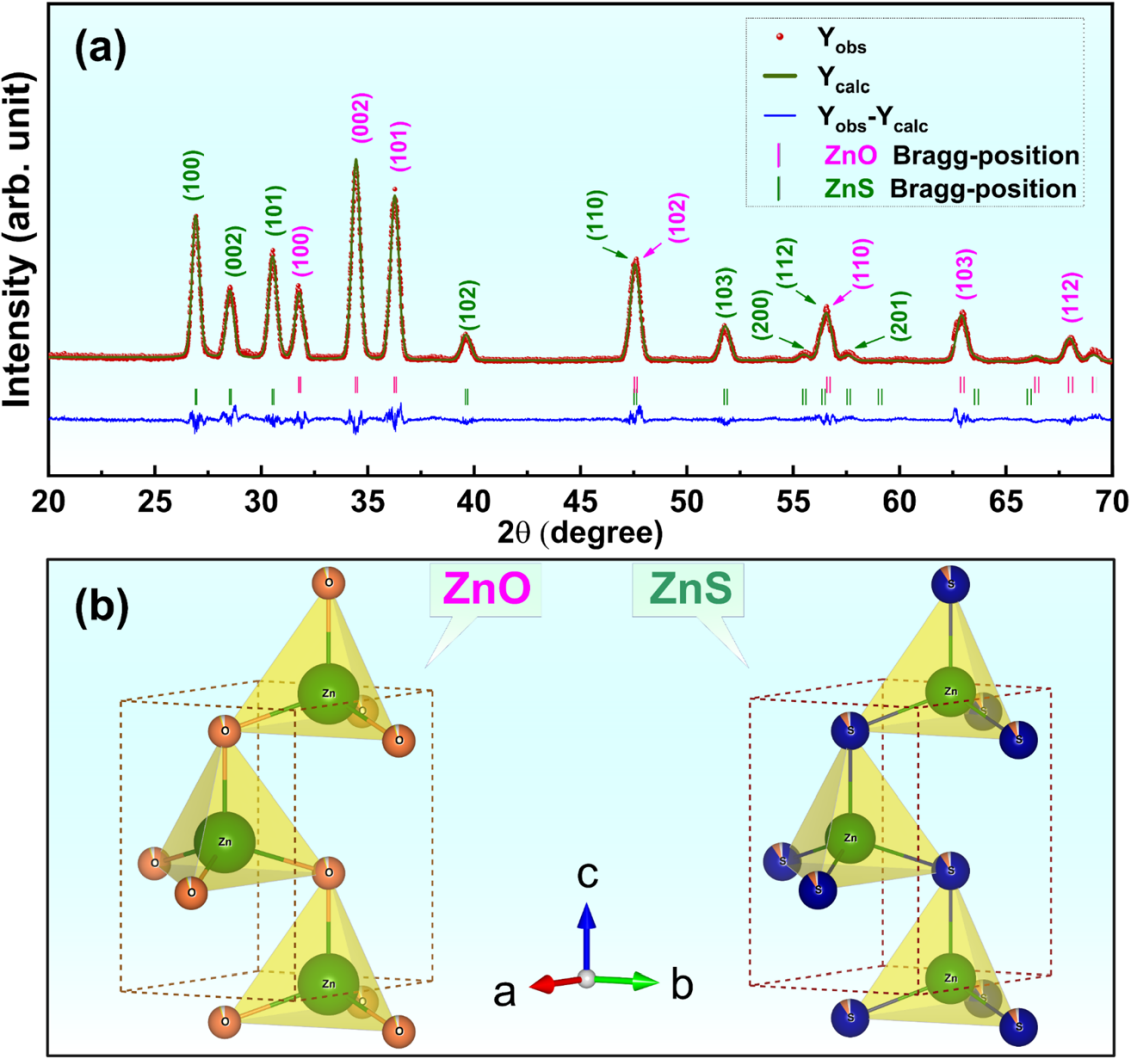
(a) XRD pattern refinement of ZnS/ZnO phosphor following grinding and post-annealing at 1000 °C for 2 h in Ar. (b) Visualization of the ZnO and ZnS crystal structure within the resulting phosphor, created using VESTA software.

Fig. 1a presents XRD pattern refinement of ZnS/ZnO phosphors received after grinding by a high-energy planetary ball milling and post-annealing at 1000 °C in Ar for 2 h.

Both ZnS and ZnO phases are evident in the XRD pattern (with ZnS, ZnO, and ZnS/ZnO annealed under the same conditions, as shown in Fig. S1†). The distinct diffraction peaks at 2*θ* = 26.9°, 28.6°, and 30.6° correspond to the (100), (002), and (101) planes of the hexagonal ZnS phase (wurtzite-2H, space *P*6_3_*mc*(186), JCPDS Card No. 01-1450).^26^ Additionally, the peaks at 2*θ* = 31.8°, 34.5°, and 36.3° are attributed to the (100), (002), and (101) planes of hexagonal zincite phase ZnO (JCPDS Card No. 36-1451).^26,27^ The crystallite sizes of the synthesized powders were calculated using the Scherrer equation, *d*_Scherrer_ = 0.9*λ*/(*β* × cos *θ*),^24^ where *λ* is the X-ray wavelength (*λ* = 1.54 Å), *θ* is the diffraction angle, and *β* is the full width at half-maximum (FWHM, in radians) of a diffraction peak. Table 1 compares the average crystallite sizes of ZnS and ZnO phases synthesized by various methods, highlighting the significant impact of fabrication techniques on structural properties. Hydrothermal synthesis produces the smallest crystallite sizes (1.58–1.97 nm for ZnS and 3.91–4.40 nm for ZnO), making it ideal for ultrafine material application.^30^ In contrast, combustion synthesis yields moderate sizes (10–11 nm),^24^ while the solid-state method results in much larger crystallites, up to 54 nm,^28^ indicating limited refinement. The solution method offers intermediate crystallite sizes (6.95 nm for ZnS and 22.65 nm for ZnO), striking a balance between refinement and structural control.^29^

**Table 1:** Comparison of the average crystallite sizes of ZnO, ZnS, and ZnO/ZnS samples determined using the Scherrer equation in this study and previously published works

Sample	Fabrication method	Crystallite size of ZnS (nm)	Crystallite size of ZnO (nm)	Ref.
ZnS/ZnO nanocomposite phosphor	Combustion synthesis	11	10	24
ZnS/ZnO nanocomposites	Solid-state method	46–50	45–54	28
ZnO–ZnS core–shell nanostructure	Solution method	6.95	22.65	29
ZnS–ZnO nanocomposites	Hydrothermal routes	1.58–1.97	3.91–4.40	30
ZnS/ZnO phosphor	High-energy planetary ball milling method and annealing	16.71	17.37	This work

Thanks to the advantage of the high-energy planetary ball milling, this study achieves relatively small sizes (16.71 nm for ZnS and 17.37 for ZnO), combining efficient synthesis with potential defect-related enhancements. These results highlight the importance of selecting appropriate synthesis methods to optimize material performance.

FullProf Suite analysis reveals that the ZnS and ZnO phases comprise 73.84% and 26.16%, respectively. Their Rietveld refinement parameters, including the reliability factors (*χ*^2^, *R*_p_, and *R*_exp_), lattice constants and unit cell volume are listed in Table 2. It is worthwhile noting that the calculated patterns are consistent with the measured data, with low *R*_p_ and *R*_exp_ values confirming the reliability of the structural models used for the refinements. Based on the lattice parameters obtained from the Rietveld refinement, a schematic of the ZnS and ZnO unit cells with wurtzite structures was generated using VESTA software, as shown in Fig. 1b. Table 2 clearly presents detailed information on lattice parameters, phase fractions, atomic positions, and occupancy values for the ZnS and ZnO phases, allowing for easy comparison. The phase fractions are accurate, summing to 100%. Lattice parameters are precise, though indicating the symmetry type (*e.g.*, cubic or hexagonal) would provide additional context. The fractional positions and Wyckoff positions are well-reported, but the rationale behind the sulfur occupancy in ZnS (0.907) could be clarified. Occupancy and atomic displacement (*U*) values are useful, though further explanation of negative or low *U* values, especially for sulfur in ZnO, would be beneficial.

**Table 2:** The obtained parameters from Rietveld's refinement of the XRD data for the ZnSO sample

Phases	Lattice parameters (Å)	Lattice volume (Å^3^)	Phase fraction (%)	Atoms	Fractional position co-ordinates			Wyckoff positions	Occupancy	*U*
					*x*	*y*	*z*			
ZnS phase	*a* = 3.82394	79.234 ± 0.019	73.84 ± 0.52	Zn	0.33333	0.66667	0.00000	2b	1.000	0.104
				S	0.33333	0.66667	0.37480	2b	0.907	0.059
	*c* = 6.25692			O	0.33333	0.66667	0.37480	2b	0.067	0.059
ZnO phase		47.642 ± 0.008	26.16 ± 0.22	Zn	0.33333	0.66667	0.00000	2b	1.000	0.076
	*a* = 3.25075			O	0.33333	0.66667	0.38200	2b	0.944	−0.020
	*c* = 5.20587			S	0.33333	0.66667	0.38200	2b	0.015	0.000
“*R*” values		*R*_p_ = 9.42%		*R*_exp_ = 8.73%				*χ* ^2^ = 2.45		

The refinement quality (*R*_p_ = 9.42%, *R*_exp_ = 8.73%, *χ*^2^ = 2.45) suggests a reasonable fit, but a brief explanation of these values would improve clarity. The low sulfur occupancy in ZnO (0.015) may indicate impurities, which should be addressed. Additionally, the table could benefit from details on the XRD data range and refinement software used for a more thorough assessment.

**Fig. 2:**
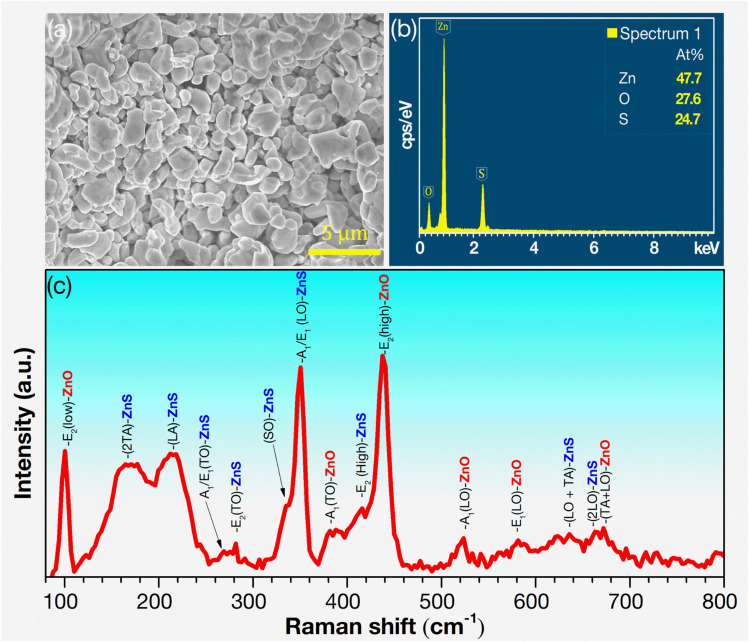
(a) FESEM image, (b) EDS spectrum, and (c) Raman spectrum of ZnS/ZnO phosphor.

Fig. 2a presents the FESEM image of ZnS/ZnO phosphor, with particle sizes ranging from 0.5 to 3 μm and clear grain boundaries. Fig. 2b shows the EDS spectrum of ZnS/ZnO phosphor powder, revealing atomic proportions of Zn (47.7%), O (27.6%), and S (24.7%). The lower S content than O may be due to the partial oxidation of ZnS into ZnO during annealing, which is consistent with the XRD phase composition results. The morphology and elemental composition of the annealed ZnS and ZnO are detailed in Fig. S2 and Table S1.†

The Raman spectrum of the ZnS/ZnO phosphor, excited by a 532 nm laser, is shown in Fig. 2c, with the resonance modes for both ZnS and ZnO summarized in Table 3. This table compares the Raman peak positions of ZnS/ZnO phosphors synthesized through various methods, highlighting differences across multiple samples. The distinctive peaks at 216.6, 269.2, 281.5, 333.2, 351.2, and 415.2 cm^−1^ correspond to the longitudinal acoustic (LA), transverse optical [A_1_/E_1_(TO)], translational oscillation [E_2_(TO)], surface optical (SO), longitudinal optical [A_1_/E_1_(LO)], and translational oscillation E_2_(high) resonance modes of ZnS, respectively.^20,31^ Additionally, the peaks at 100.6, 382.0, and 438.5 cm^−1^, corresponds to the E_2_(low), A_1_(TO), and E_2_(high) resonance modes of ZnO.^40,42^ Notably, the sharp peaks at 351.2 cm^−1^ (A_1_/E_1_(LO) mode of ZnS) and 438.5 cm^−1^ (E_2_(high) mode of ZnO) confirm the presence of wurtzite ZnS and ZnO phases.^32^ These results demonstrate the effectiveness of this synthesis method in capturing a broad range of Raman modes, providing valuable insights into the material's vibrational properties. The absence of certain peaks in some references (indicated by dashes) may suggest variations in synthesis conditions or material quality.

**Table 3:** Comparison on Raman modes of ZnS/ZnO phosphor and previous reports

Materials	Synthesis method	Raman peak position (cm^−1^)								
		E_2_(low)	LA	A_1_/E_1_ (TO)	E_2_ (TO)	SO	A_1_/E_1_(LO)	A_1_ (TO)	E_2_(high)/LO + TA	Ref.
ZnS:Al^3+^ nanorods	Hydrothermal method	—	—	251.1	—	—	346.8	—	422.5	31
ZnS–ZnO composites	Solution combustion	—	—	—	—	—	—	—	429	32
ZnS nanoribbons	Hydrothermal method	—	218	262	—	—	351	—	424	33
ZnS nanowires	Thermal evaporation	—	—	269	282	—	350	—	—	34
ZnS micro–nanostructures	Hydrothermal method	—	—	250	271	335	350	—	420	35
ZnS nanostructures	Thermal evaporation	—	—	267	280	336.2	347.7	—	—	36
ZnS/ZnO nanostructure	Chemical colloidal process	—	—	—	—	—	350	—	434	37
ZnO nanoparticles	Sol–gel method	98	—	—	—	—	339	—	437	38
ZnO:Al thin film	Magnetron sputtering	101	—	275	—	—	—	—	450	39
ZnO:Al nanoparticles	Sol–gel method	—	—	—	—	—	330	373	433	40
ZnO:N nanostructures	Microwave irradiation	—	—	271	—	—	—	380	437	41
ZnS/ZnO	High-energy planetary ball milling and post-annealing	100.6	216.6	269.2	281.5	333.2	351.2	382.0	415.2 (ZnS)	This work
									438.5 (ZnO)	

### 3.1. Binding energy analysis

**Fig. 3:**
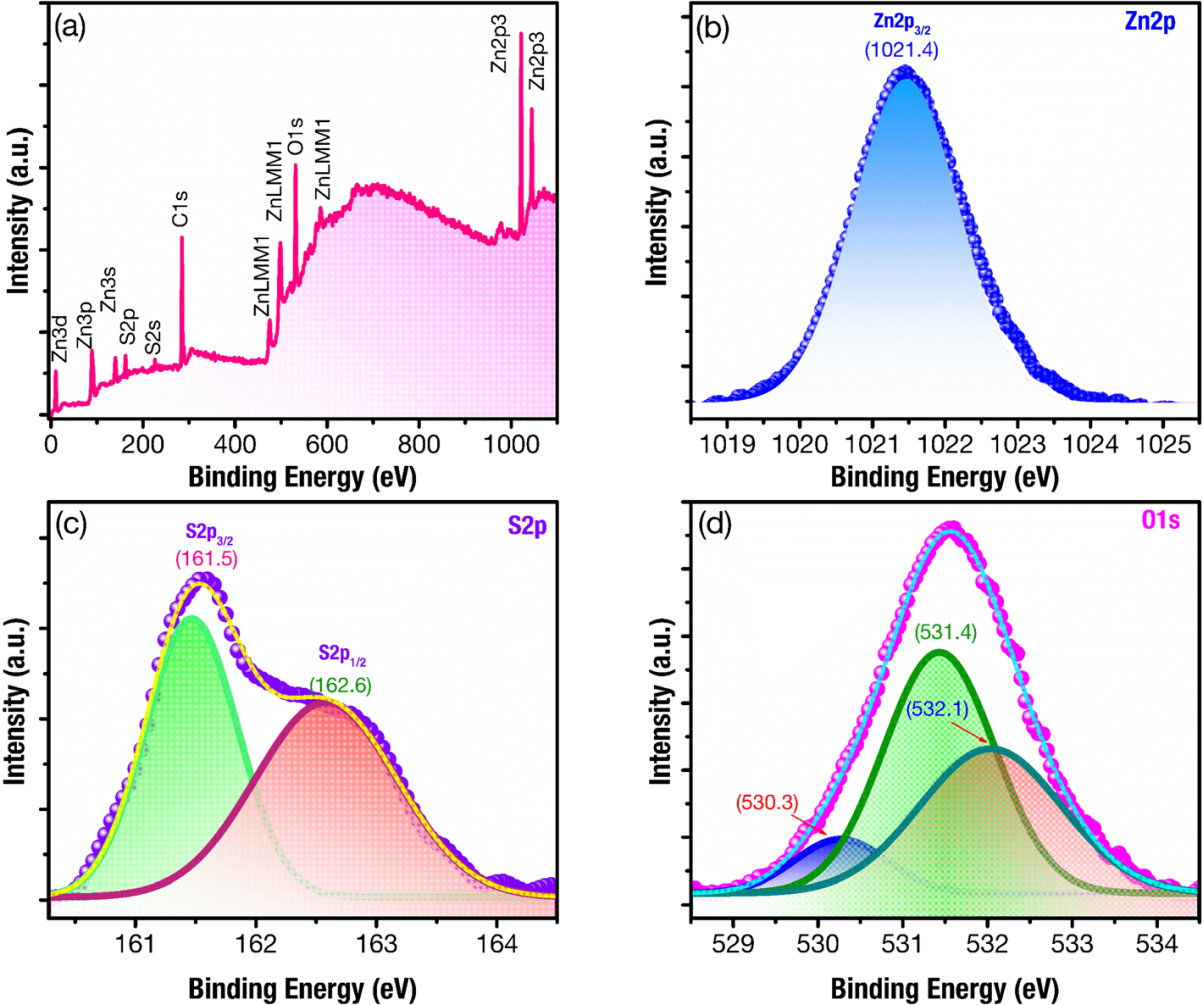
XPS spectra of ZnS/ZnO phosphor powder: (a) complete survey, and their high-resolution binding energy spectra of (b) Zn 2p (c) S 2p, (d), and O 1s.

Fig. 3a shows the XPS survey spectrum of ZnS/ZnO phosphor powder, with clear peaks corresponding to Zn, O, S, and C. The XPS spectra are normalized according to the C 1s binding energy peak at 284.65 eV.^43,44^ In Fig. 3b, the high-resolution binding energy spectrum of Zn 2p exhibits a well-defined Zn 2p_3/2_ peak at approximately 1021.4 eV, aligning with the binding energy of Zn^2+^ in ZnO and ZnS.^45,46^ The deconvolution of the S 2p XPS spectra (Fig. 3c) shows two peaks at 161.5 eV and 162.6 eV, corresponding to the S 2p_3/2_ and S 2p_1/2_ electronic states, with the S 2p_3/2_ binding energy indicating Zn–S bonding.^43,47–49^

The analysis of the O 1s XPS spectra, using multiple Gaussian fitting, reveals three peaks at 530.3, 531.4, and 532.1 eV (Fig. 3d). The peak at 530.3 eV can be attributed to the lattice oxygen in ZnO,^39,50^ while the peak at 531.4 eV is assigned to the O^2−^ ions in oxygen-deficient regions within ZnO matrix, indicating the presence of oxygen vacancy (V_O_).^39,51,52^ The peak at 532.1 eV may be associated with H_2_O or O_2_ absorbed on the surface.^41,50^ These findings suggest a significant presence of defects related to oxygen vacancies within the host lattice of ZnS/ZnO phosphor.

### 3.2. UV-vis spectroscopy analysis and photoluminescence study

**Fig. 4:**
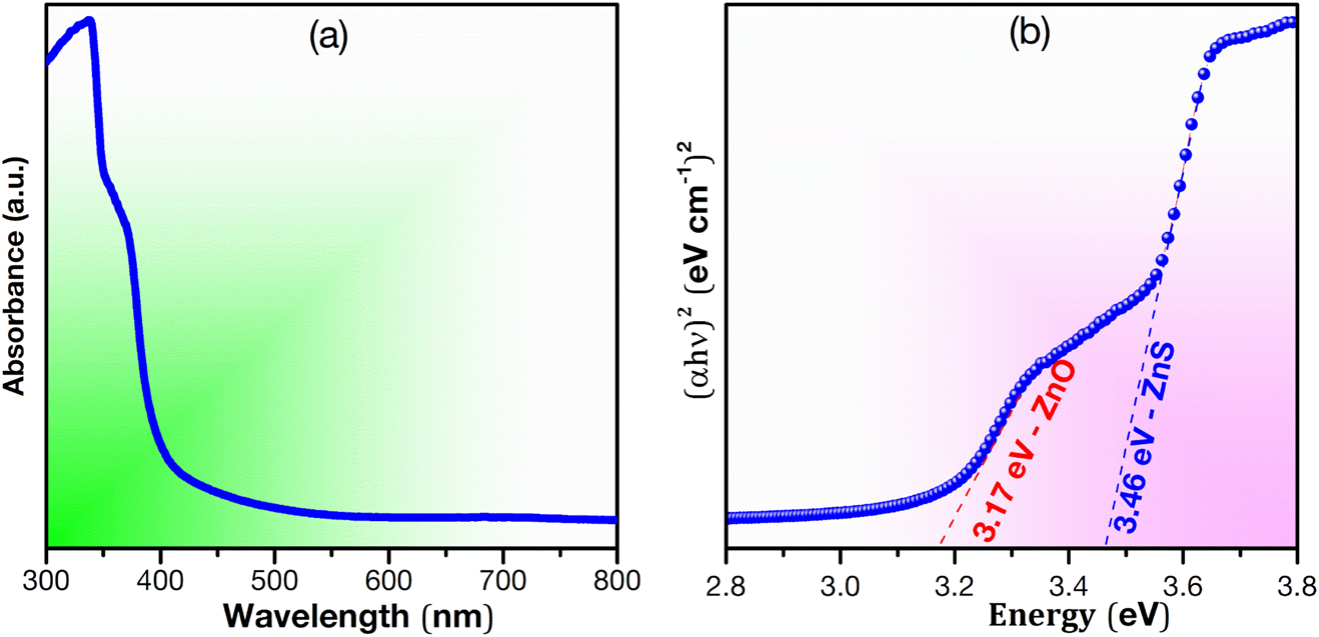
(a) UV-vis spectroscopy analysis of ZnS/ZnO sample and (b) Tauc plot.

DRS UV-visible analysis was used to examine the optical properties of the synthesized ZnS–ZnO sample (Fig. 4), focusing on reflectance calculated using the Kubelka–Munk equation. Two distinct optical bands were observed, corresponding to ZnS (3.46 eV) and ZnO (3.17 eV). The bandgap of ZnS/ZnO was compared to that of ZnS and ZnO annealed under the same conditions (see Fig. S3 and Table S2†). The reduced bandgap in ZnS/ZnO may be attributed to surface defects or diffusion of S into ZnO and O into ZnS.^29,53^

**Fig. 5:**
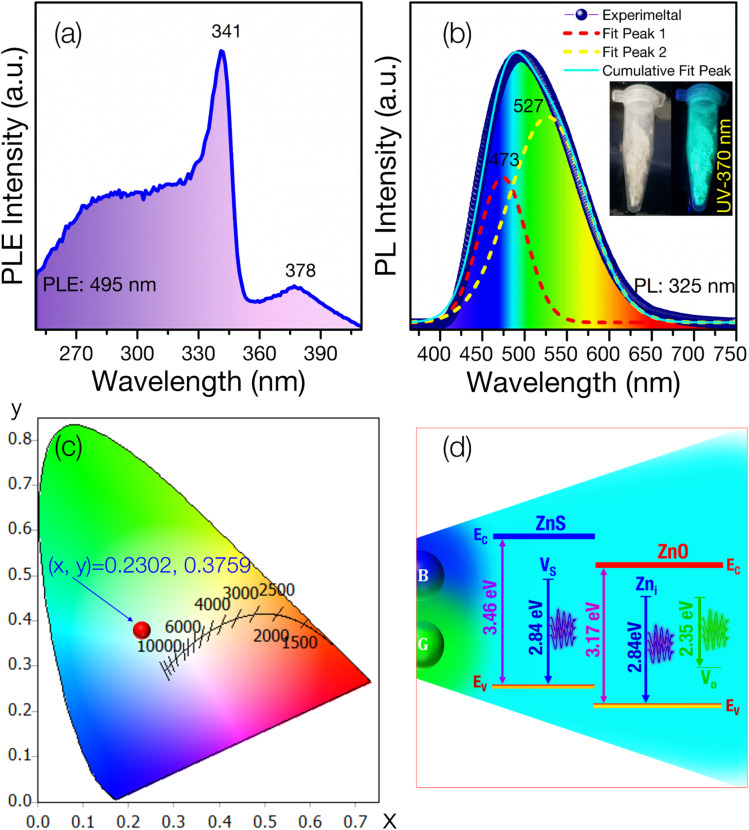
(a) PLE and (b) PL spectrum of ZnS/ZnO phosphor excited by the 325 nm wavelength of Xe lamp; (c) CIE chromaticity diagram of the ZnS/ZnO phosphors (*λ*_ex_ = 325 nm) and (d) proposed mechanism for emitting cyan emission from ZnS/ZnO phosphor.

Fig. 5a presents the PLE spectrum of ZnS/ZnO phosphor monitored at 495 nm. The spectrum shows a characteristic absorption band of ZnS peaking at 341 nm (∼3.63 eV) and a weaker ZnO absorption band centered at 378 nm (∼3.28 eV). This effect may be due to the high-energy planetary ball milling and annealing at elevated temperatures, which likely induce intrinsic defects in the ZnS and ZnO host lattices^25^ or result in the formation of ZnS/ZnO heterostructures.^28^ These findings are consistent with Raman and XPS analyses.

Fig. 5b illustrates the PL spectrum of ZnS/ZnO phosphor, exhibiting a broad visible emission (400–700 nm) with a peak at 495 nm under 325 nm Xe lamp excitation. Gaussian deconvolution reveals two prominent peaks at 473 nm (blue) and 527 nm (green), with green emission dominating the PL spectrum. The blue peak at 473 nm is attributed to intrinsic defects, such as sulfur vacancies (V_S_) in ZnS^54,55^ or zinc interstitials (Zn_i_) in ZnO.^43,56,57^

The green emission at 527 nm is likely due to oxygen vacancies (V_O_) in ZnO^55,58^ or the ZnO/ZnS interface.^59^ Using OSRAM Sylvania's ColorCalculator software, the CIE 1931 color coordinates of the ZnS/ZnO phosphor were calculated to be (0.2302, 0.3759), placing it in the cyan emission region (Fig. 5c). Fig. 5d depicts the energy level diagram, illustrating the combination of blue and green emissions to produce cyan light.

**Fig. 6:**
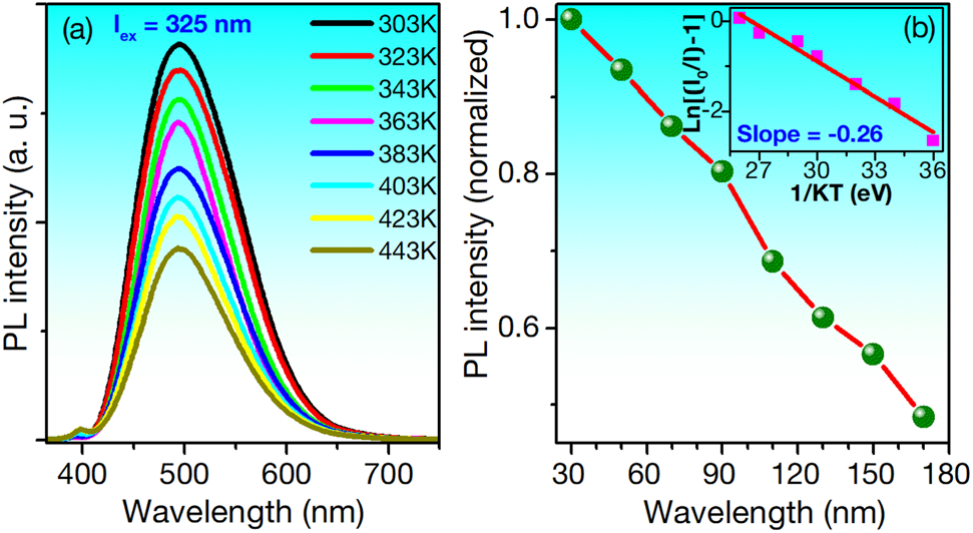
(a) Temperature-dependent emission spectra excited by the wavelength of 235 nm and (b) the dependence of normalized PL emission intensity of spectra of ZnS/ZnO phosphor and temperature. The insets illustrate the relationship between ln[(*I*_0_/*I*) − 1] and 1/*kT*.

The thermal stability of phosphors is critical for LED applications, as operational temperatures can exceed 150 °C.^22^Fig. 6a presents the PL spectra of ZnS/ZnO phosphor at various temperatures, excited at 325 nm.

While the spectra maintain their shape in the visible region, the intensity decreases as the temperature rises from 303 to 443 K. At 423 K, approximately 51% of the initial intensity is retained. For comparison, the Ca_3_Y(PO_4_)_3_:Eu^2+^, Mn^2+^ phosphor retains 52% under similar conditions,^60^ while the Ba_3_Y_2_B_6_O_15_:Ce^3+^, Tb^3+^ phosphor retains only 21.9%.^1^ The thermal quenching effect is linked to the activation energy (Δ*E*), calculated using the modified Arrhenius eqn (1):^22^

1 *I*_T_ = *I*_0_[1 + *A* exp(−Δ*E*/*kT*)]^−1^

where *I*_0_ and *I*_T_ are the initial PL intensity and the PL intensity at a specific temperature, respectively. A is a constant, and *k* = 8.62 × 10^−5^ eV K^−1^ is Bolzmann's constant. By fitting the plot of ln[*I*_0_/*I*_T_ − 1] *versus* 1/*kT*, Δ*E* was determined as 0.26 eV (Fig. 6b). This value could be compared with some phosphors using for WLED such as Ca_3_Y(PO_4_)_3_:Eu^2+^, Mn^2+^ (0.166 eV),^60^ Ca_4_ZrGe_3_O_12_:Bi^3+^ (0.228 eV),^2^ Ba_3_Bi(PO_4_)_3_: Sm^3+^, Eu^3+^ (0.21 eV),^61^ and NaGd_9_(SiO_4_)_6_O_2_:Bi^3+^ (0.296 eV).^62^

The electroluminescence (EL) spectrum of the NUV 370 nm chip driven at 500 mA in Fig. S5,† highlighting a strong NUV emission peak at 370 nm. The phosphor-converted cyan LEDs exhibit a broad visible emission region and a weaker NUV peak. The visible emissions are produced by the phosphor layer excited by the 370 nm NUV chip, while the residual NUV emission in the EL spectrum originates from unabsorbed light from the NUV chip. Quantum efficiency (QE) is a critical parameter for assessing the suitability of phosphors for LED applications.^63^ The QE of the ZnS/ZnO phosphor, when coated onto a NUV chip, can be calculated using the following eqn (2):^22^2
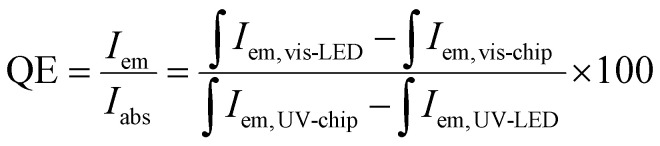
where, ∫*I*_em,vis-LED_ and ∫*I*_em,vis-chip_ correspond to the integrated visible emission of the LED with and without the phosphor layer, respectively.

Using this equation, the QE of the ZnS/ZnO phosphor was estimated to be around 77%. This QE value surpasses those of several other reported materials, including ZnO nanowires (20%),^64^ Ba_3_Lu_2_B_6_O_15_:Ce^3+^,Tb^3+^ phosphor (51%),^65^ Mn-doped ZnS/ZnO nanobelts (61%),^66^ RbNa_1-*y*_(Li_3_SiO_4_)_2_:*y*Eu^2+^ phosphor (62.9%),^11^ and it is comparable to Ca_2_GdHf_2_(AlO_4_)_3_:Ce^3+^ (80%).^67^

**Fig. 7:**
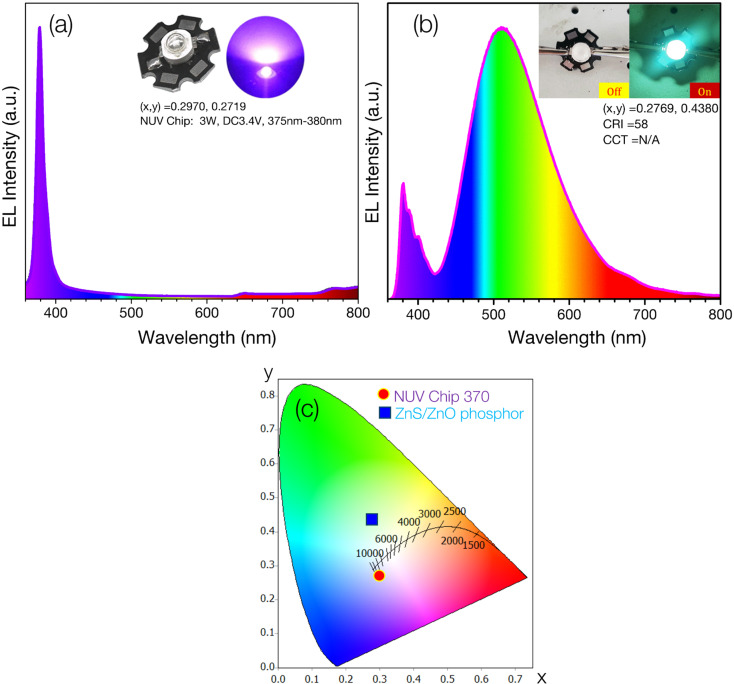
(a) EL spectrum of NUV chip (370 nm, 3 W, 3.4 V); (b) EL spectra of NUV-pumped ZnS/ZnO phosphor; (c) the CIE 1931 chromaticity diagram of the fabricated cyan-LED and NUV-chip.

The digital image and electroluminescence (EL) parameters of the NUV chip are presented in the inset of Fig. 7a. The EL spectrum of the NUV chip, driven by a 500 mA current, reveals strong NUV emission with chromaticity coordinates (*x*, *y*) of (0.2970, 0.2719), spanning the 350-410 nm range and peaking at approximately 377 nm. Fig. 7b shows the EL spectrum of the ZnS/ZnO phosphor-coated NUV chip, accompanied by a digital image of the phosphor-coated NUV chip under 500 mA current and no current.

The emitted light is biased toward cyan, demonstrating the ZnS/ZnO phosphor's ability to convert NUV to visible cyan light. The measured chromaticity coordinates (*x*, *y*) and color rendering index (CRI) of the cyan-LED are (0.2769, 0.4380) and 58, respectively. Fig. 7c displays the CIE chromaticity coordinates for both the NUV chip and the ZnS/ZnO phosphor-coated NUV chip. The color coordinate discrepancy between the phosphor-coated NUV-LED and the phosphor powder is likely due to visible emission contributions from the NUV chip. Table 4 provides a comprehensive comparison of several cyan-emitting phosphors.

**Table 4:** Excitation source, color coordinates, quantum efficiency, and activation energy of several cyan-emitting phosphors

Phosphor powder	Synthesis method and experimental conditions	Strongest emission peak (nm)	FWHM (nm)	Highest absorption peaks (nm)	Color-coordinates	QE (%)	Lifetime (μs)	Activation energy, *E*_a_ (eV)	Ref.
ZnS–ZnO	Hydrothermal, 120 °C, 24 h, ambient	∼470	—	316–319	—	—	—	—	30
ZnS:KBr,Mn^2+^	Solid-state, 750–1100 °C, 2 h, CO ambient	468–476	—	344	—	—	1.35–2.47 × 10^3^	—	68
ZnO:Er^3+^/Ho^3+^	Solid-state, 500 °C, 2 h	377	—	502	—	—	8.47–27.5	—	69
ZnO:Eu^3+^,Dy^3+^	Precipitation at 80 °C, calcination for 2 h at 700 °C	482	—	390	(0.514, 0.395)	—	—	—	70
CaZnOS:Bi^3+^,Mn^2+^	Solid-state, 1100 °C, 3 h	485–491	—	375	(0.350, 0.323)	—	—	—	71
ZnO	Hydrothermal, 200 °C, 4 h	520	—	355	(0.302, 0.488)	—	—	—	72
ZnO:Ce^3+^	Solid-state, 1000 °C, 6 h	499	—	375	(0.23, 0.33)	—	—	—	73
ZnS	Solid-state, 1050 °C, N_2_/H_2_ ambient	460	—	342	—	—	—	—	74
ZnO:Al^3+^									22
ZnS/ZnO	High-energy planetary ball milling, 1000 °C, 2 h	495		341, 378	(0.277, 0.438)	77		0.26	This work

This table details their synthesis methods, excitation sources, emission peaks, color coordinates, quantum efficiency (QE), and activation energy. The data highlights a variety of synthesis techniques and their impact on the material's optical properties. Notably, the ZnS/ZnO phosphor in this work exhibits a strong emission peak at 495 nm, a quantum efficiency of 77%, and an activation energy of 0.26 eV, which demonstrates excellent performance compared to other reported materials. The inclusion of these metrics strengthens the understanding of the relationship between synthesis conditions and phosphor properties.

## 4. Conclusions

In summary, the cyan-emitting phosphor derived from ZnS/ZnO has been successfully synthesized using a high-energy planetary ball milling method combined with post-annealing. The ZnS/ZnO phosphor, with particle sizes ranging from 1 to 3 μm, exhibits cyan emission with CIE chromaticity coordinates of (*x*, *y*) = (0.2302, 0.3759). A prototype near-ultraviolet (NUV)-pumped cyan LED was also developed, showing chromatic coordinates of (0.2769, 0.4380) and a quantum efficiency of 77%, achieved by coating the NUV chip at 370 nm with ZnS/ZnO phosphor. The phosphor exhibits strong thermal stability with an activation energy of 0.26 eV. These findings open new avenues for cyan LED development using ZnS/ZnO phosphor, offering substantial promise for phosphor-converted white LEDs (pc-WLEDs) with full-color capability.

## Data availability

The data supporting this article have been included as part of the ESI.†

## Conflicts of interest

The authors declare no conflict of interest.

## Supplementary Material

RA-015-D4RA08038F-s001
