# Supplementary material for: Highly efficient and thermally stable cyan-emitting ZnS/ZnO phosphors for full-visible-spectrum LED lighting
Source: RSC Adv. 2025 Jan 7;15(1):616–27. doi: 10.1039/d4ra08038f (PMC11704878; doi:10.1039/d4ra08038f)
Supplement: RA-015-D4RA08038F-s001 [file RA-015-D4RA08038F-s001.pdf]

Supplementary Information (SI) for RSC Advances.
This journal is © The Royal Society of Chemistry 2024

Supplement Information

Highly efficient and thermally stable cyan-emitting ZnS/ZnO phosphors for full-visible-spectrum LED lighting

Do Quang Trung[a,*], Manh Trung Tran[b,*], Nguyen Van Du[a], Nguyen Duy Hung[c], Tong Thi Hao Tam[d], Nguyen Tu[a], Pham Minh Tri[b], Hoang Gia Chuc[b], Nguyen Duc Trung Kien[e], Nguyen Van Quang[f], Pham Thanh Huy[b]

[a]Faculty of Fundamental Sciences, Phenikaa University, Yen Nghia, Ha-Dong district, Hanoi 10000, Vietnam

[b]Faculty of Materials Science and Engineering, Phenikaa University, Yen Nghia, Ha-Dong district, Hanoi 10000, Vietnam

[c]Faculty of Electronic Materials and Components, School of Materials Science and Engineering, Hanoi University of Science and Technology (HUST), No. 01 Dai Co Viet, Hanoi 10000, Vietnam

[d]School of Information Technology and Digital Economics (SITDE), National Economics University (NEU), 207 Giai Phong Street, Hanoi 10000, Vietnam

[e]Faculty of Electrical and Electronic Engineering, Phenikaa University, Yen Nghia, Ha-Dong district, Hanoi 10000, Vietnam

[f]Department of Chemistry, Hanoi Pedagogical University 2, Phuc Yen, Vinh Phuc, Vietnam

* Corresponding author:

Email address: trung.doquang@phenikaa-uni.edu.vn and trung.tranmanh@phenikaa-uni.edu.vn;

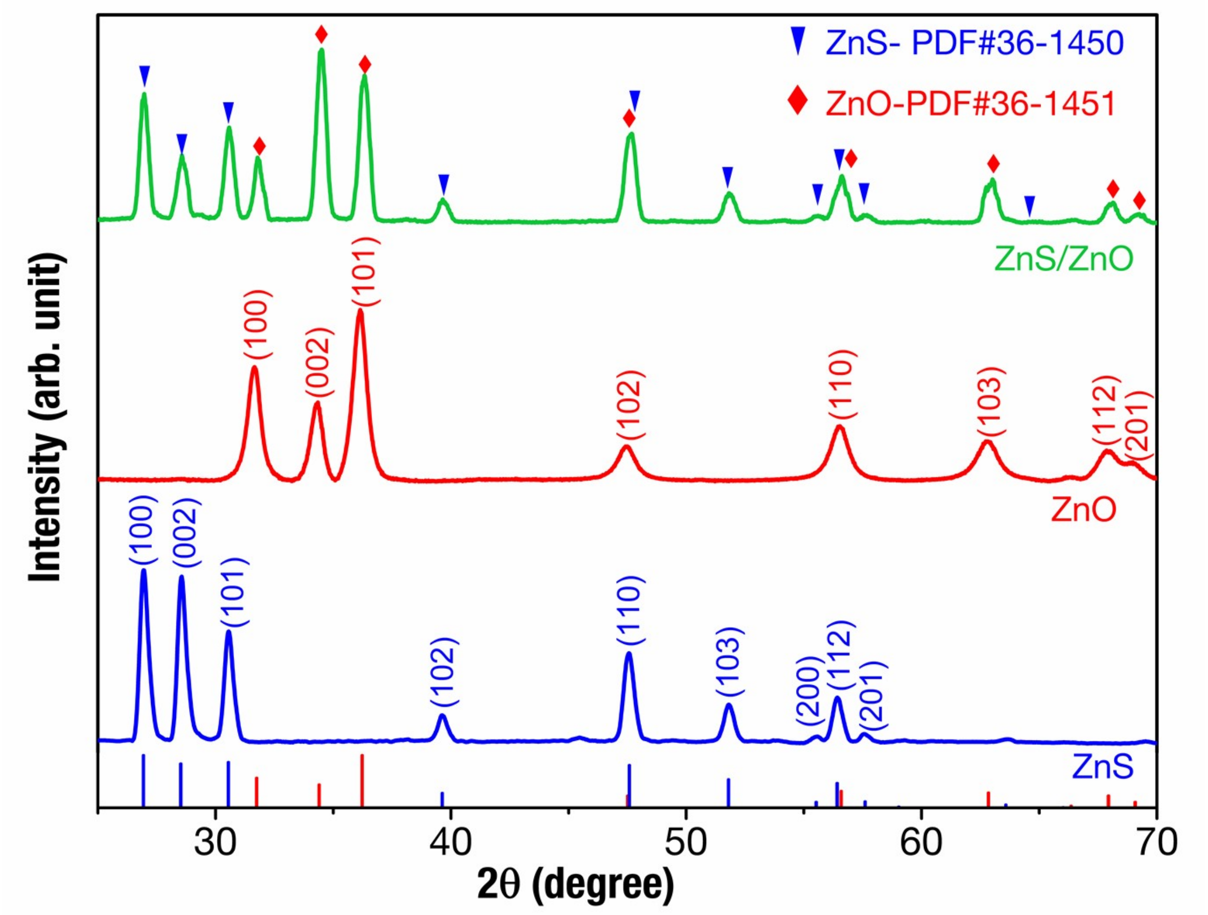

Fig. S1. The XRD patterns of the commercial ZnS, ZnO, and ZnS/ZnO phosphor powders anneal at 1000 °C for 2 hours in an Ar gas environment.

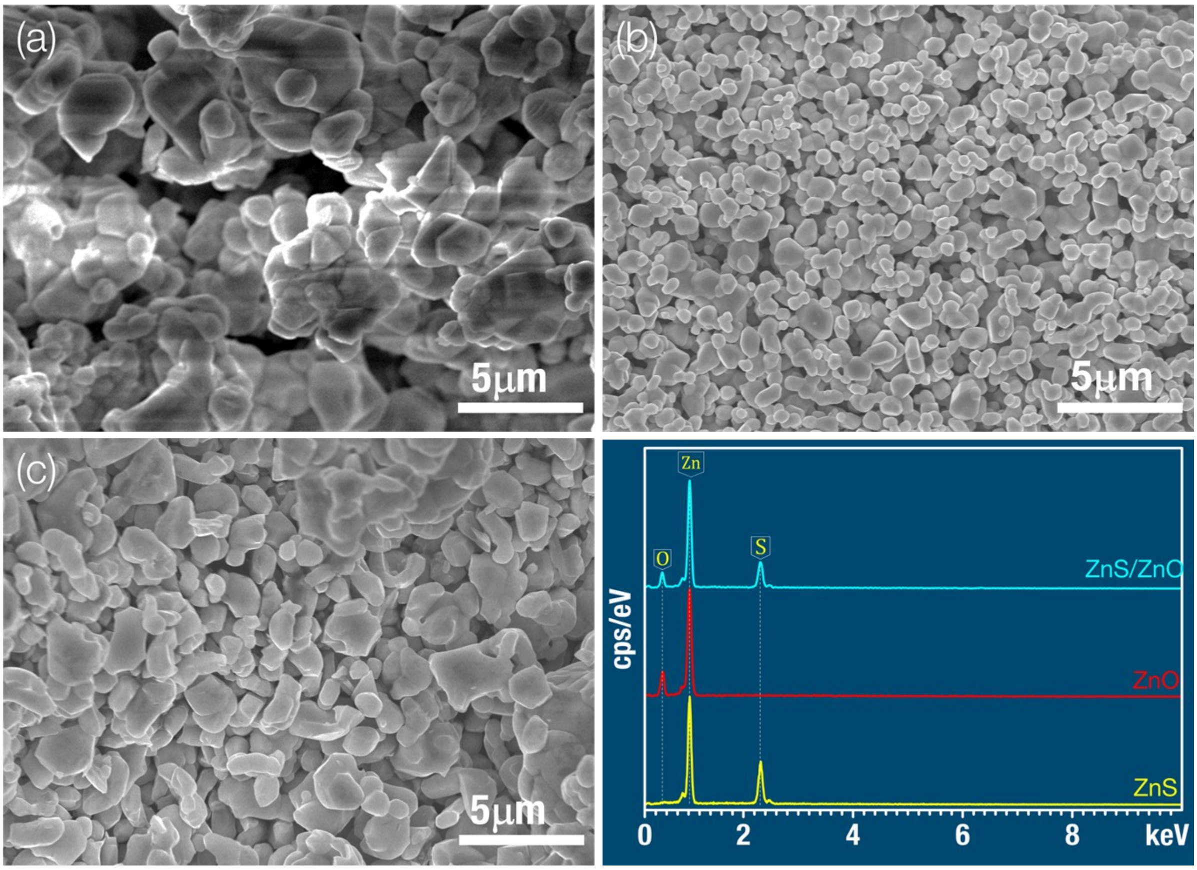

Fig. S2. The FESEM images of (a) ZnS, (b) ZnO, (c) ZnS/ZnO annealed at 1000 °C for 2 hours in an Ar gas environment, and (d) their EDS spectra, respectively.

Table S1. Atomic ratio of Zn, S, O elements in ZnS, ZnO and ZnS/ZnO samples

Elements	ZnS	ZnO	ZnS/ZnO
**Zn**	51.1	57.8	47.7
**S**	47.5	42.2	24.7
**O**	1.4	--	27.6

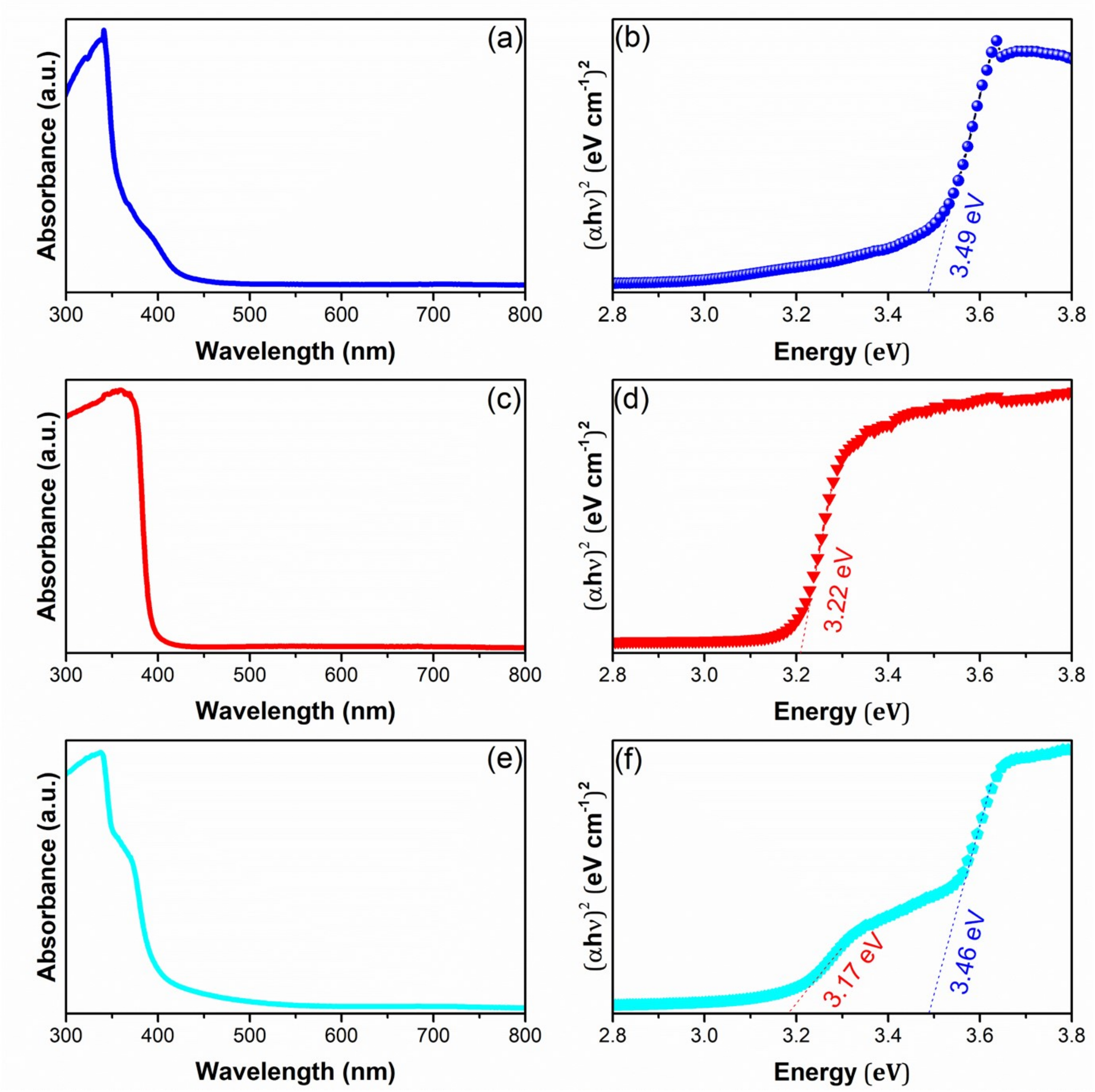

Fig. S3. UV-Vis spectra of ZnS, ZnO, and ZnS/ZnO, along with their Tauc plots of $(\alpha h\nu)^2$ versus E.

Table S2 presents the bandgap values of ZnS, ZnO, and ZnS/ZnO phosphors derived from Fig. S3. The bandgap of the ZnS phase is 3.49 eV, while the ZnO phase shows a bandgap of 3.22 eV. For the ZnS/ZnO composite, the ZnS and ZnO phases exhibit bandgaps of 3.46 eV and 3.17 eV, respectively, indicating slight modifications in the bandgap of each phase within the composite material.

Table S2. The bandgap of ZnS, ZnO, and ZnS/ZnO phosphors derived from Fig. S3

Samples / Bandgap (eV)	ZnS	ZnO	ZnS/ZnO
**ZnS phase**	3.49	--	3.46
**ZnO phase**	--	3.22	3.17

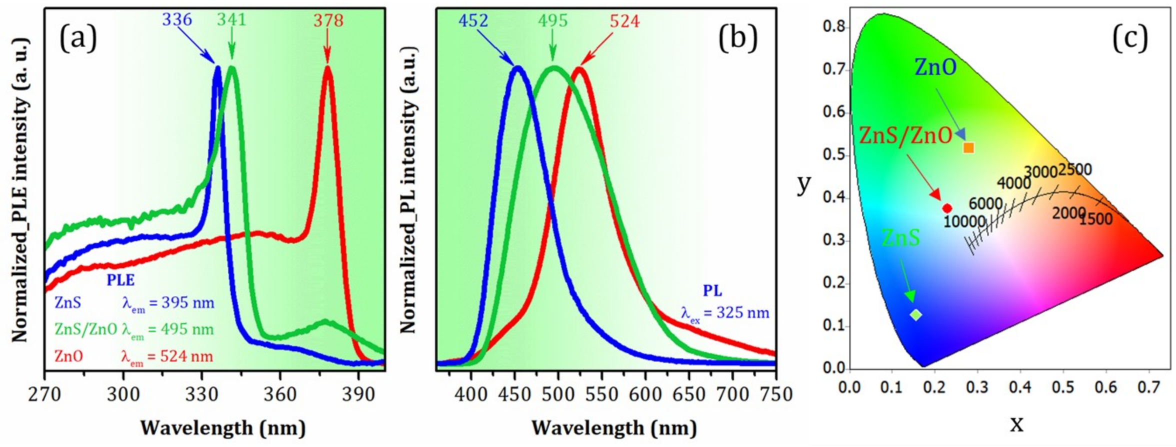

Fig. S4. (a) PLE and (b) PL spectra of ZnS, ZnO, and ZnS/ZnO phosphors; (c) Corresponding CIE 1931 chromaticity diagram for ZnS, ZnO, and ZnS/ZnO phosphors.

Table S3. Optical parameters of ZnS/ZnO, ZnS, and ZnO samples annealed under identical conditions

Sample	x	y	CRI	CCT
ZnS	0.1556	0.1267	--	>100000
ZnO	0.2792	0.5191	44	--
ZnS/ZnO	0.23002	0.3760	50	--

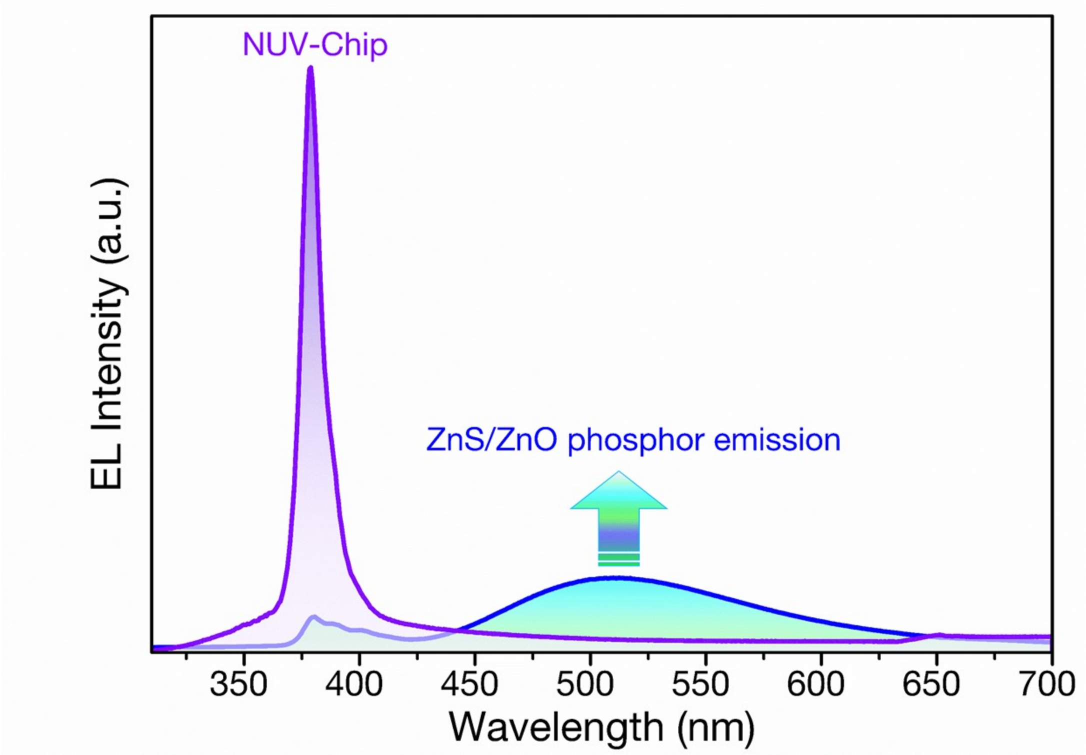

Fig. S5. EL spectrum of NUV (370 nm) chip and NUV-pumped ZnS/ZnO phosphor-coated NUV chip.
